# Associations Between Delivery Mode and Early Childhood Body Mass Index Z-Score Trajectories: A Retrospective Analysis of 2,685 Children From Mothers Aged 18 to 35 Years at Delivery

**DOI:** 10.3389/fped.2020.598016

**Published:** 2020-12-17

**Authors:** Lihong Zhang, Liuxia Huang, Zhiyuan Zhao, Renjuan Ding, Hongnian Liu, Wenchao Qu, Xiao Jia

**Affiliations:** ^1^Key Laboratory of Birth Regulation and Control Technology of National Health Commission of China, Shandong Maternal and Child Health Development Research Center, Maternal and Child Health Care Hospital, Shandong University, Jinan, China; ^2^Department of Pediatrics, The Huangdao Maternal and Child Health Care Hospital, Qingdao, China; ^3^Department of Pharmacy, The Binhai Central Health Center of Huangdao, Qingdao, China; ^4^Department of Pharmacy, The Huangdao Maternal and Child Health Care Hospital, Qingdao, China; ^5^Department of Orthopedic, The Binhai Central Health Center of Huangdao, Qingdao, China; ^6^Department of Traditional Chinese Medicine, The Huangdao Maternal and Child Health Care Hospital, Qingdao, China

**Keywords:** delivery mode, cesarean delivery, childhood, body mass index, growth trajectory

## Abstract

**Objective:** To investigate the association between cesarean delivery (CD) and trajectory patterns of age- and sex-specific body mass index (BMI) z-score in early childhood.

**Methods:** A retrospective cohort study was conducted among 2,685 children whose maternal age at the time of birth was between 18 and 35 years, and birth data and anthropometric measurement data during their ages 3–60 months were collected. A group-based trajectory modeling approach was used to identify distinct BMI z-score trajectories, and multinomial logistic regressions were applied to estimate the associations among CD (both elective and non-elective combined), elective and non-selective CD, and BMI z-score trajectory classes.

**Results:** Of the 2,685 participants, 46.5% (*N* = 1,248) were born by vaginal delivery (VD), 20.7% (*N* = 556) by elective CD, and 32.8% (*N* = 881) by non-elective CD. Five BMI z-score trajectory patterns were identified, and they were “increasing from moderate to high” (10.1%, *n* = 270), “increasing from mild to moderate” (34.2%, *n* = 919), “increasing from low to high” (10.5%, *n* = 283), “stable mild” (30.1%, *n* = 808), and “stable low” (15.1%, *n* = 405) groups. Compared with children delivered by VD, those who delivered by CD (both elective and non-elective combined), elective CD, and non-elective CD were associated with the “increasing from moderate to high” trajectory [odds ratio (OR) = 1.61, 95% confidence interval (CI): 1.13–2.29; OR = 1.64, 95%CI: 1.06–2.54; and OR = 1.59, 95%CI: 1.05–2.39, respectively] and were also associated with the “increasing from low to high” trajectory (OR = 1.60, 95%CI: 1.17–2.19, OR = 1.75, 95%CI: 1.16–2.63; and OR = 1.53, 95%CI: 1.00–2.34, respectively).

**Conclusion:** Both elective and non-elective CD were associated with the risk of accelerated weight gain in early childhood.

## Introduction

The cesarean delivery (CD) rate has increased significantly in the past decades worldwide (1). Ranking as one of the countries with the highest CD rate, China has witnessed a more rapid increase in CD rate, which increased from 28.8% in 2008 to 36.7% in 2018 with an annual percentage change of 1.8% (2). CD could be life-saving for both the mothers and their offspring if appropriately performed and medically indicated, which should only be performed when a vaginal delivery (VD) places the mothers or newborns at risk because of medical reasons (3). However, many Chinese mothers chose a CD without any medical necessities for the reasons of fearing pain by VD, perceiving CD safer than VD, choosing a lucky day as babies' birthdate, and obstetricians' beliefs or attitudes toward CD (4, 5). Given the high rate of CD, especially elective CD, the potential adverse impact of CD on mother and offspring has been concerning, and in comparison with VD, higher risk of maternal death or infection, neonatal respiratory morbidity, and adverse effects to subsequent pregnancies have been identified (6). In addition, CD may result in altered neonatal gut microbiota and thus pre-dispose offspring to an increased risk of childhood overweight and obesity (7).

Childhood overweight and obesity have become a global public health concern, and numerous studies have shown that there is a distinct rise in overweight and obesity prevalence in both urban and rural regions of China (8). Childhood overweight and obesity are associated with short-term risk of cardiovascular risk factors [such as elevated blood pressure (9), adverse lipid profiles (10)]. Noteworthily, there is accumulating evidence suggesting that childhood obesity tends to track into adulthood and subsequently increase the long-term risk of adult cardiovascular and metabolic diseases (11, 12).

Some studies have investigated the association between CD and childhood obesity. Although some studies have reported that CD is significantly associated with risk of childhood overweight/obesity or higher body mass index (BMI) (13–15), heterogeneous results presenting null associations have also been observed (16). In addition, studies exploring the association between CD subtypes (elective and non-elective CD) and indicators of childhood obesity also reported inconsistent results (17, 18). Furthermore, most existing studies focused on BMI in a single or limited number of measurements, ignoring its dynamic changes during a specific period.

In response to these literature gaps, we designed a retrospective cohort study to investigate the association between CD as well as its subtypes (elective and non-elective CD) and trajectory patterns of age- and sex-specific BMI z-score in children aged 3–60 months, which could overcome the limitation of static analysis and increase comparability across ages.

## Methods

### Study Population

We designed this retrospective cohort study in Huangdao Maternal and Child Health Care Hospital, which provides prenatal care, delivery, postnatal, and child health care service for all residents in Huangdao district, Qingdao, China. This study is based on the National Basic Public Health Service Project (http://www.nbphsp.org.cn/jbgw/06rt/), which was launched by the Chinese government in 2009. In this project, one of the nine service categories was providing health services for Chinese children aged 0–6 years for free, including the establishment of maternal and children health records, post-partum visits, physical examinations, and health consultation. Health providers involved in this project were all trained according to national standards and required to provide elaborate health services for children who were at ages of 3, 6, 8, 12, 18, 24, 30, 36, 48, 60, and 72 months.

Participants were singleton offspring born at term in this hospital between January 1, 2012, and December 31, 2013, and had height/length and weight repeatedly measured 4 to 10 times during the following 5 years. Inclusion criteria were (1) singleton offspring born with 37–42 completed weeks of gestation and (2) mother's age at birth between 18 and 35 years. Exclusion criteria were (1) mothers who were already diagnosed with types 1 or 2 diabetes before the index pregnancy or (2) offspring conceived by *in vitro* fertilization, born with congenital disabilities or postnatal diseases that could interfere with body composition development, or (3) offspring with missing covariate data. The study was conducted in accordance with the Declaration of Helsinki, and the protocol and the use of the database were approved by the Ethics Committee of the Huangdao Maternal and Child Health Care Hospital.

### Exposure and Covariates

Delivery mode (VD, elective CD, or non-elective CD) data and the following individual-level covariates were collected from the birth records: maternal age at birth, maternal education level, parity, hypertensive disorders of pregnancy, gestational diabetes mellitus, gestational age at birth, and birth weight. Maternal BMI was measured at the first prenatal visit, and BMI status was defined by using Asian BMI cut points (underweight: BMI <18.5, normal: 18.5 ≤ BMI <23.0, and overweight/obesity: BMI ≥ 23.0) (19).

### Outcomes

Age- and sex-specific BMI z-score trajectory patterns were the outcomes of interest, and anthropometric measurements were performed by trained health providers for participants dressed in light indoor clothing without shoes. Recumbent length for children aged <24 months and standing height for children aged ≥24 months were measured to the nearest 0.1 cm with an infantometer and a stadiometer, respectively. Body weight was measured to the nearest 0.1 kg using a digital scale. All of the instruments were calibrated before measuring, and both length/height and weight were measured twice; then, the mean values were recorded. BMI was calculated as weight (kilogram) divided by height/length (square meter), and age- and sex-specific BMI z-score was calculated using the “igrowup”package for SAS based on World Health Organization Child Growth Standards (20).

### Statistical Analysis

A group-based trajectory modeling approach was applied to identify trajectories of BMI z-score from aged 3 months through 60 months. This approach has been widely used in other childhood populations across longitudinal data to identify several discrete classes and individuals in each class following similar trends of change (21). To fully reflect the potential BMI z-score trajectories, only an individual having BMI measured at least four time points out of ages of 3, 6, 8, 12, 18, 24, 30, 36, 48, and 60 months could be included in this analysis. Analyses were conducted via PROC TRAJ macro in SAS (22), and the time scale was age at anthropometric measurement. Repeated trajectory analyses were conducted to identify the latent classes by setting the number of groups from 2 to 7, and model fit was assessed based on the following criteria: Bayesian Information Criterion, the mean posterior probability for group membership >0.7, and clinical plausibility and the interpretability of the classes (23, 24). Once the optimal number was determined, linear, quadratic, and cubic terms were tested to determine the appropriate shape of each trajectory (22). Then, participants were assigned to the trajectory group for which they had the greatest posterior predictive probability.

Differences in maternal and offspring characteristics among the identified BMI z-score trajectory groups were examined using one-way analysis of variance or chi-square-test. To examine the association between delivery mode and BMI z-score trajectory patterns, crude and adjusted multinomial logistic regression models were used to report OR with 95% CI. We constructed three models: model 1 was the crude model, model 2 adjusted for gestational age at birth and birth weight, and model 3 adjusted for gestational age at birth, birth weight, maternal age at birth, maternal education level, maternal BMI at the first prenatal visit, parity, hypertensive disorders of pregnancy, and gestational diabetes mellitus. We further conducted a stratification analysis by child sex to test whether there is a sex-specific effect on BMI z-score trajectory patterns. All statistical analyses were conducted using SAS version 9.4 (SAS Institute Inc., Cary, NC, USA).

## Results

### Study Population Characteristics

**Figure 1:**
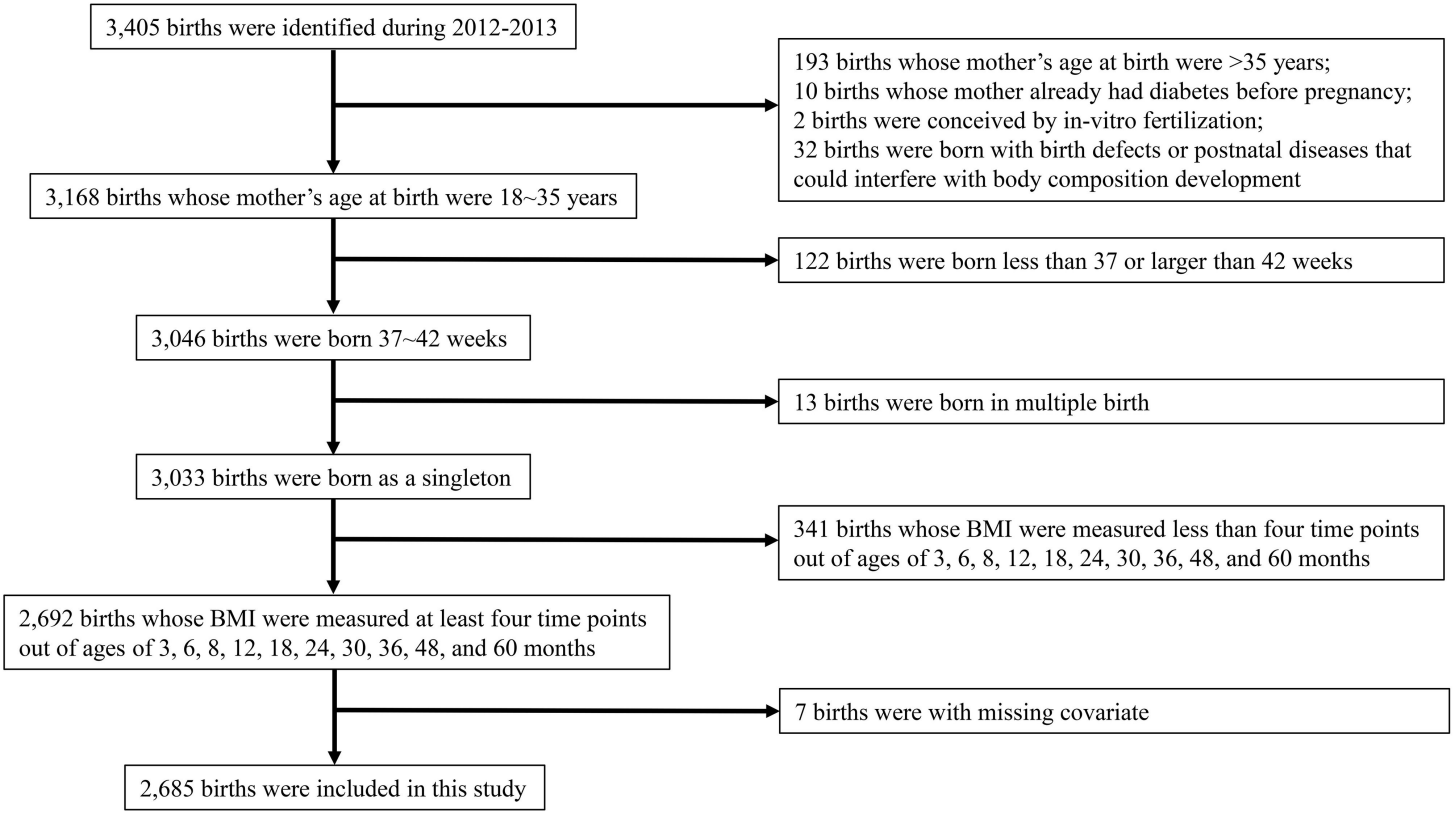
Flow chart describing the selection process of subjects for the present study.

A total number of 3,405 birth records in the years 2012 and 2013 were retrieved from the birth records, and 2,685 offspring were included in this study according to the inclusion and exclusion criteria. The characteristics of the studied mother–child pairs are shown in Table 1 and the flow chart in Figure 1. In this analysis population, 46.5% (*N* = 1,248) of the children were born by VD, 20.7% (*N* = 556) by elective CD, and 32.8% (*N* = 881) by non-elective CD. The mean maternal age at birth was 28.2 years, and the prevalence of maternal obesity/overweight was 29.8%. In addition, 52.3% (*N* = 1,404) of the children were boys, and the mean gestational age and birth weight were 39.1 weeks and 3.06 kg, respectively. We compared the characteristics of the excluded children and the children having <4 anthropometric measures with the characteristics of the included children, respectively (Supplementary Table 1). Excluded children tended to be from older mothers and having lower birth weight, and children having <4 anthropometric measures tended to be from mothers with lower education level.

**Table 1:** Participant characteristics for each of the five different BMI z-score trajectory groups.

	**Overall**	**Increasing from moderate to high**	**Increasing from mild to moderate**	**Increasing from low to high**	**Stable mild**	**Stable low**	***P*-value^**†**^**
	**(*n* = 2,685)**	**(*n* = 270)**	**(*n* = 918)**	**(*n* = 283)**	**(*n* = 808)**	**(*n* = 406)**	
**Maternal characteristics**							
**Deliver mode**							
VD	1,248 (46.5)	99 (36.7)	455 (49.6)	98 (34.6)	404 (50.0)	192 (47.3)	<0.001^‡^
CD (both elective and non-elective combined)	1,437 (53.5)	171 (63.3)	463 (50.4)	185 (65.4)	404 (50.0)	214 (52.7)	
Elective CD	558 (20.8)	71 (26.3)	184 (20.0)	80 (28.3)	162 (20.0)	61 (15.0)	
Non-elective CD	879 (32.7)	100 (37.0)	279 (30.4)	105 (37.1)	242 (30.0)	153 (37.7)	
Maternal age (years)	28.2 ± 4.1	28.5 ± 4.6	28.4 ± 4.0	28.2 ± 4.7	28.1 ± 3.8	27.8 ± 3.9	0.090
**Maternal BMI status**							
Underweight	186 (6.9)	9 (3.3)	59 (6.4)	20 (7.1)	40 (5.0)	58 (14.3)	<0.001
Normal	1,699 (63.3)	167 (61.6)	557 (60.6)	192 (67.8)	533 (66.0)	250 (61.7)	
Obesity/overweight	800 (29.8)	95 (35.1)	303 (33.0)	71 (25.1)	234 (29.0)	97 (24.0)	
**Maternal education level**							
≤ 9 years	386 (14.4)	23 (8.5)	105 (11.4)	52 (18.4)	124 (15.3)	82 (20.2)	<0.001
>9 years	2,299 (85.6)	247 (91.5)	814 (88.6)	231 (81.6)	684 (84.7)	323 (79.8)	
**Hypertensive disorders of pregnancy**							
No	2,509 (93.4)	246 (91.1)	849 (92.4)	268 (94.7)	761 (94.2)	385 (95.1)	0.122
Yes	176 (6.6)	24 (8.9)	70 (7.6)	15 (5.3)	47 (5.8)	20 (4.9)	
**Gestational diabetes mellitus**							
No	2,272 (84.6)	209 (77.4)	739 (80.4)	261 (92.2)	692 (85.6)	371 (91.6)	<0.001
Yes	413 (15.4)	61 (22.6)	180 (19.6)	22 (7.8)	116 (14.4)	34 (8.4)	
**Parity**							
Primiparous	2,429 (90.5)	241 (89.3)	798 (86.8)	271 (95.8)	741 (91.7)	378 (93.3)	<0.001
Multiparous	256 (9.5)	29 (10.7)	121 (13.2)	12 (4.2)	67 (8.3)	27 (6.7)	
**Offspring characteristics**							
Gestational age (weeks)	39.1 ± 1.7	39.8 ± 1.8	39.5 ± 1.7	38.5 ± 1.6	38.9 ± 1.5	38.6 ± 1.7	<0.001
Birth weight (g)	3,061 ± 411	3,469 ± 362	3,213 ± 347	2,807 ± 353	3,030 ± 339	2,682 ± 302	<0.001
**Sex**							
Female	1,281 (47.7)	114 (42.2)	430 (46.8)	139 (49.1)	419 (51.9)	179 (44.2)	0.023
Male	1,404 (52.3)	156 (57.8)	489 (53.2)	144 (50.9)	389 (48.1)	226 (55.8)	

Values express n (%) or mean ± standard deviation.

BMI z-score, age- and sex-specific body mass index z-score; VD, vaginal delivery; CD, cesarean delivery.

† P-values from ANOVA F-tests (comparisons of means) and from Chi-square-tests of independence (comparison of proportions).

‡ *This P-value for comparing proportions between VD and CD, and VD, elective CD, and non-elective CD are all <0.001*.

### Group-Based Trajectory Modeling

**Figure 2:**
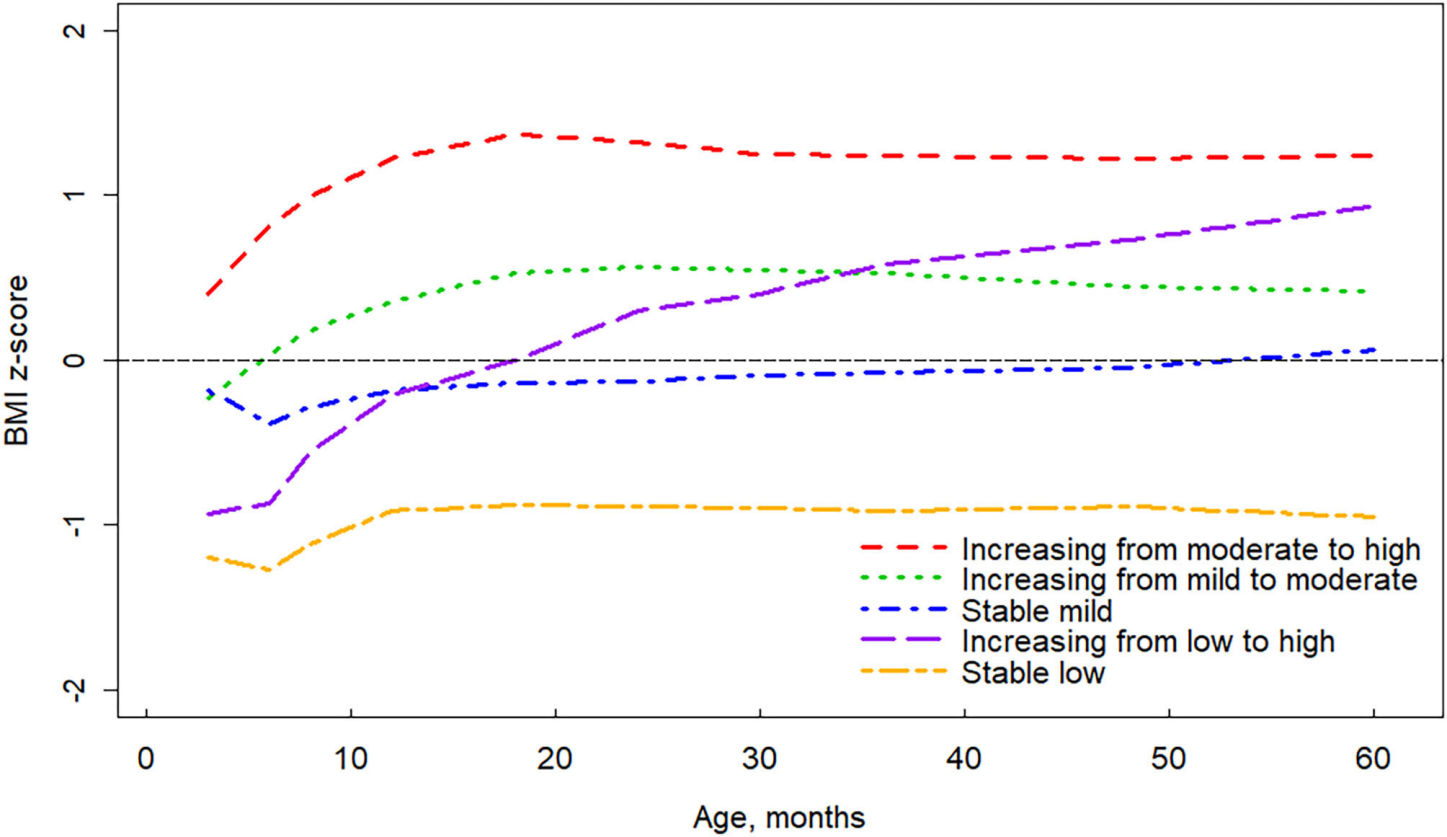
Trajectories of age- and sex-specific body mass index z-score for children aged 3 to 60 months.

Children had a median of six repeated anthropometric measurements (interquartile range: 5–8 times) from 3 to 60 months of age. Numbers of children who had anthropometric measurements at the age of 3, 6, 8, 12, 18, 24, 30, 36, 48, and 60 months were 2,136, 2,029, 1,767, 1,938, 1,713, 1,725, 1,608, 1,627, 1,534, and 1,489, respectively. The five-group model proved optimal for describing BMI z-score trajectory patterns from age 3 months through 60 months (Figure 2). We labeled the five trajectories as “increasing from moderate to high” (10.1%, *n* = 270), “increasing from mild to moderate” (34.2%, *n* = 919), “increasing from low to high” (10.5%, *n* = 283), “stable mild” (30.1%, *n* = 808), and “stable low” (15.1%, *n* = 405) groups. Among the five groups, significant differences in proportions of delivery mode, maternal BMI status, maternal education level, gestational diabetes mellitus, parity, offspring sex, and means of gestational age and birth weight were observed (Table 1).

### Association of Delivery Mode With Body Mass Index z-Score Trajectory Groups

In the unadjusted model, compared with children delivered by VD, those who delivered by CD were significantly associated with the two trajectories characterized by accelerated BMI z-score gain (for “increasing from moderate to high” group: OR = 1.73, 95%CI: 1.29–2.32, for “increasing from low to high” group: OR = 1.89, 95%CI: 1.41–2.53), and results were similar for both elective and non-elective CD (Table 2). In addition, the non-elective CD was significantly associated with the “stable low” group (OR = 1.34, 95%CI: 1.03–1.79). After adjustment for potential confounding factors representing maternal and offspring characteristics, compared with children delivered by VD, those who delivered by CD (both elective and non-elective combined), elective CD, and non-elective CD were still significantly associated with the risk of having the trajectory of “increasing from moderate to high” group (OR = 1.61, 95%CI: 1.13–2.29; OR = 1.64, 95%CI: 1.06–2.54; and OR = 1.59, 95%CI: 1.05–2.39, respectively) and the trajectory of “increasing from low to high” group (OR = 1.60, 95%CI: 1.17–2.19; OR = 1.75, 95%CI: 1.16–2.63, respectively; OR = 1.53, 95%CI: 1.01–2.34, respectively). In the stratification analysis by child sex (Supplementary Table 2), the effect estimates were similar to the main results, revealing no sex-specific effect on BMI z-score trajectory patterns.

**Table 2:** Crude and multivariable-adjusted odds ratios (OR) and 95% confidence intervals (CI) for offspring BMI z-score trajectories associated with cesarean delivery as well as its subtypes vs. vaginal delivery.

**Variable**		**Increasing from moderate to high**	**Increasing from mild to moderate**	**Increasing from low to high**	**Stable low**
**Model 1**					
VD		1.00 (Reference)	1.00 (Reference)	1.00 (Reference)	1.00 (Reference)
CD (both elective and non-elective combined)		1.73 (1.29–2.32)	1.02 (0.84–1.24)	1.89 (1.41–2.53)	0.90 (0.70–1.15)
Elective CD		1.79 (1.23–2.59)	1.00 (0.77–1.30)	2.04 (1.42–2.92)	0.79 (0.55–1.11)
Non-elective CD		1.67 (1.21–2.35)	1.03 (0.82–1.28)	1.81 (1.30–2.51)	1.34 (1.03–1.79)
**Model 2**					
VD		1.00 (Reference)	1.00 (Reference)	1.00 (Reference)	1.00 (Reference)
CD (both elective and non-elective combined)		1.65 (1.20–2.26)	0.97 (0.79–1.19)	1.75 (1.27–2.41)	0.88 (0.67–1.16)
Elective CD		1.70 (1.12–2.58)	0.95 (0.72–1.26)	1.86 (1.26–2.76)	0.76 (0.51–1.11)
Non-elective CD		1.61 (1.14–2.27)	0.99 (0.78–1.25)	1.68 (1.16–2.44)	1.30 (0.99–1.70)
**Model 3**					
VD		1.00 (Reference)	1.00 (Reference)	1.00 (Reference)	1.00 (Reference)
CD (both elective and non-elective combined)		1.61 (1.13–2.29)	0.93 (0.74–1.18)	1.60 (1.17–2.19)	0.85 (0.64–1.12)
Elective CD		1.64 (1.06–2.54)	0.92 (0.68–1.24)	1.75 (1.16–2.63)	0.73 (0.50–1.08)
Non-elective CD		1.59 (1.05–2.39)	0.94 (0.73–1.21)	1.53 (1.01–2.34)	1.25 (0.93–1.67)

BMI z-score, age- and sex-specific body mass index z-score; VD, vaginal delivery; CD, cesarean delivery.

All of the four BMI z-score trajectory groups were compared to the “stable mild” group.

*Model 1 was the crude model; Model 2 was adjusted for gestational age at birth and birth weight; Model 3 was adjusted for gestational age at birth, birth weight, maternal age at birth, maternal education level, maternal BMI at the first prenatal visit, parity, hypertensive disorders of pregnancy, and gestational diabetes mellitus*.

## Discussion

In this retrospective cohort study, we identified five distinct BMI z-score trajectories during early childhood and found significant associations of CD and its subtypes with two trajectories characterized by accelerated BMI z-score gain. Our findings suggest that newborns delivered by CD, irrespective of subtypes, would have an accelerated weight growth pattern during early childhood. To the best of our knowledge, this is the first study to explore the association between delivery mode and BMI z-score trajectory patterns during early childhood. This study provides new insights into the origins of overweight and obesity in early childhood life and emphasizes the importance of reducing the overused cesarean section.

In this population, the prevalence of maternal obesity/overweight is 29.8%, which is similar to the prevalence of obesity/overweight in women aged 20–39 years from the 2011 China Health and Nutrition Survey (26.3%) (25). A total of 53.5% of children were delivered by CD during 2012–2013 in this hospital from an eastern coastal district, which was higher than the mean CD rate in China in 2013 (34.6%) (26). However, there was distinct geographic variation in CD rates across China, and the CD rates of Shanghai and Hangzhou (two eastern coastal cities) were also larger than 50% during 2012–2013 (26). All of these regions ranked in the highest tertile economic level in China, and the expanded access to hospital care and financial incentives for physicians to perform cesareans may lead to a higher CD rate (26).

Our association analyses showed that children delivered by CD were associated with an increased risk of having trajectories characterized by accelerated weight gain, although the effect estimates attenuated distinctly after adjustment for maternal and offspring variables. This result is similar to previous studies reporting positive associations between CD and larger childhood BMI or risk of overweight/obesity (13–15). However, two cohort studies revealed inconsistent results. Pei et al. reported a significant association between CD and childhood obesity risk only at 2 years (OR = 1.68, 95% CI: 1.10–2.58) but not at age 6 or 10 years in a German cohort (27). Vinding et al. (28) reported a higher mean BMI at 6 months (β = 0.37, 95% CI: 0.14–0.60) in children delivered by CD but not at 5 or 13 years in two Danish cohorts. In contrast, one meta-analysis found that CD was associated with overweight and obesity not only among children (3–8 years) but also in adolescence (9–18 years) and adult (≥19 years) populations (29).

With regard to the effects of different subtypes of the CD on offspring overweight and obesity, existing studies also showed discordant findings. Both elective and non-elective CDs were found to be associated with obesity in children aged 3–6 years from China (18) and in children aged 7 years from the United States (17). However, Cai et al. (30) reported that only elective CD, but not non-elective CD, was associated with risk of overweight in Singaporean children aged 12 months, and Smithers et al. (16) found elective CD was not associated with BMI z-score among Australian children aged 3–6 years. Differences in age groups of subjects and definition of outcomes of interest in analytic models among the studies mentioned earlier may explain the discrepant results. Moreover, smaller sample size and CD and obesity rates in some studies may also reduce the power to detect a significant association between CD and outcomes.

The group-based trajectory modeling approach in our study makes it possible to explore the potential heterogeneity of BMI z-score changes during early childhood, which is an essential difference against the studies mentioned earlier. Several previous studies have identified the role of stable high BMI or rapid BMI gain during childhood contributing to increased risk for obesity in adulthood, suggesting the potential ability of childhood BMI trajectories in predicting health outcomes in later life (23, 31). These findings underline the necessity to explore influencing factors leading to those seemingly unhealthy BMI trajectories.

The exact mechanisms of CD leading to obesity or accelerated BMI gain during childhood is still unclear, but alterations in the gut microbiota via different delivery modes are a potential biological mechanism speculated by many researchers. The fetus and the intrauterine environments are believed to be sterile, and thus, the intestinal microbiome developed beginning at birth may be dependent on whether the newborn's first contact was with the vaginal canal or skin microbiota (17). Several studies have reported differences in gut microbiota between overweight or obese children with children in a normal weight status (32, 33). Bacteroides genera, which could protect against overweight by influencing gut energy harvest from the diet (12), were found to be lower in offspring delivered by CD compared with their VD counterparts (34). In addition, mothers who experienced a cesarean operation generally have higher odds of breastfeeding initiation failure or shorter breastfeeding duration, which has been proven to be a risk factor for childhood obesity (35). Therefore, lower Bacteroides genera and poorer maternal breastfeeding practices attributed to CD may be associated with a higher risk of abnormally accelerated growth trajectory. However, the lack of these data impeded us from exploring the exact mechanisms. In summary, the fat and glucose metabolism of offspring may be modified due to the earlier mentioned influencing factors either independently or in conjunction with each other, followed by disturbing their appetite regulation or metabolic control, and thus, the BMI growth trajectory pattern will be altered (27).

This study has several strengths. The retrospective cohort design with sufficient repeated anthropometric measurements and the group-based trajectory modeling approach enabled us to classify children into diverse growth trajectories. Besides, BMI z-score other than BMI was used to model the growth trajectory, which allowed for estimating how individuals tracked according to their rank order within BMI distributions and exempting the need for age and sex adjustment and also providing the most meaningful measure to investigate the relationship between delivery mode and change in weight status (36).

### Limitations

First, the development of obesity in childhood is a multifactorial phenomenon, but data on some confounding factors, such as maternal passive smoking, maternal breastfeeding pattern, child daily diet structure, physical and sedentary activity, and family income level, are absent in this study (35, 37, 38). However, several previous studies presenting both unadjusted and adjusted results revealed that adjustment for some environmental (antenatal active or passive smoking), breastfeeding (duration of breastfeeding), and economic (family income) factors had only a slight influence on the estimated effects (18, 30). Therefore, the impact of these unmeasured confounding factors on our results may be minimal. Second, we did not model trajectories for boys and girls separately, given that the growth curves were already adjusted by sex. Third, we included mothers only aged 18–35 years at the time of birth, and older mothers were not included in this study. However, advanced maternal age is defined as childbearing in a woman more than 35 years of age, and this is a well-established risk factor for neonatal morbidity and future childhood development (39, 40). Therefore, we excluded women >35 years of age at delivery aiming to control the complicated effects of advanced maternal age. Lastly, this study collected samples only from one hospital in a Chinese coastal district, and ~90% of the mothers were primiparous; thus, we cannot rule out the possibility that our results might be biased and not be generalizable to a population in other regions. Further prospective multiple-center studies collecting more information on maternal and offspring characteristics, such as diet, feeding practices, and environmental and economic factors that may impact the infant/child's growth trajectory, are warranted.

## Conclusion

The control of CD rate, especially the elective CD rate, should be considered as a priority goal for reducing childhood obesity and subsequently decreasing the risk of cardiovascular and metabolic diseases in later life. Communities, antenatal care sections, and hospitals should promote effective health education for pregnant women.

## Data Availability Statement

The data analyzed in this study is subject to the following licenses/restrictions: The data underlying the study are not available to the public as the project does not meet the budget requiring data sharing. Requests to access these datasets should be directed to Xiao Jia, jiaxiao2019@163.com.

## Ethics Statement

The studies involving human participants were reviewed and approved by The Ethics Committee of the Huangdao Maternal and Child Health Care Hospital.

## Author Contributions

LZ, WQ, and XJ conceptualized the study and drafted the article. LZ, LH, and RD collected all data with initial pre-processing. HL, ZZ, and LZ analyzed data. ZZ and LZ also made contributions to revising the manuscript. All authors approved the final submitted and published versions.

## Conflict of Interest

The authors declare that the research was conducted in the absence of any commercial or financial relationships that could be construed as a potential conflict of interest.
