# Supplementary material for: Associations Between Delivery Mode and Early Childhood Body Mass Index Z-Score Trajectories: A Retrospective Analysis of 2,685 Children From Mothers Aged 18 to 35 Years at Delivery
Source: Front Pediatr. 2020 Dec 17;8:598016. doi: 10.3389/fped.2020.598016 (PMC7774081; doi:10.3389/fped.2020.598016)
Supplement: Supplementary file 1 [file Table_1.DOCX]

**Table S1.** Comparison of participants that were included/excluded and included/ having <4 anthropometric measures.

|  | Children included  (n=2,685) | Children  excluded  (n=720) | P-value^†^ | Children having <4 anthropometric measures  (n=341) | P-value^†^ |
| --- | --- | --- | --- | --- | --- |
|  |  |  |  |  |  |
| **Maternal** **characteristics** |  |  |  |  |  |
| Deliver mode |  |  |  |  |  |
| VD | 1248 (46.5) | 336 (46.7) |  | 163 (47.7) |  |
| CD (both elective and non-elective combined) | 1437 (53.5) | 384 (53.3) | 0.929^‡^ | 178 (52.2) | 0.645^‡^ |
| Elective CD | 556 (20.7) | 127 (17.6) | 0.130^*^ | 64 (18.7) | 0.703^*^ |
| Non-elective CD | 881 (32.8) | 257 (35.7) |  | 114 (33.4) |  |
| Maternal age (years) | 28.2±4.1 | 28.6±4.3 | 0.011 | 28.3±4.1 | 0.336 |
| Maternal BMI status |  |  |  |  |  |
| Underweight | 186 (6.9) | 56 (7.8) | 0.443 | 31 (9.1) | 0.222 |
| Normal | 1699 (63.3) | 465 (64.6) |  | 219 (64.2) |  |
| Obesity/Overweight | 800 (29.8) | 199 (27.6) |  | 91 (26.7) |  |
| Maternal education level |  |  |  |  |  |
| ≤9 years | 386 (14.4) | 122 (16.9) | 0.086 | 68 (19.9) | 0.007 |
| > 9 years | 2299 (85.6) | 598 (83.1) |  | 273 (80.1) |  |
| Hypertensive disorders of pregnancy |  |  |  |  |  |
| No | 2509 (93.4) | 681 (94.6) | 0.265 | 318 (92.4) | 0.456 |
| Yes | 176 (6.6) | 39 (5.4) |  | 23 (7.6) |  |
| Gestational diabetes mellitus |  |  |  |  |  |
| No | 2272 (84.6) | 618 (85.8) | 0.419 | 290 (85.0) | 0.837 |
| Yes | 413 (15.4) | 102 (14.2) |  | 51 (15.0) |  |
| Parity |  |  |  |  |  |
| Primiparous | 2429 (90.5) | 636 (88.3) | 0.090 | 304 (89.1) | 0.439 |
| Multiparous | 256 (9.5) | 84 (11.7) |  | 37 (10.9) |  |
| **Offspring characteristics** |  |  |  |  |  |
| Gestational age (weeks) | 39.1±1.7 | 39.0±2.2 | 0.076 | 39.0±1.9 | 0.156 |
| Birth weight (g)^**^ | 3061±411 | 2967±423 | <0.001 | 3032±385 | 0.108 |
| Sex |  |  |  |  |  |
| Female | 1281 (47.7) | 348 (48.3) | 0.766 | 164 (48.1) | 0.894 |
| Male | 1404 (52.3) | 372 (51.7) |  | 177 (51.9) |  |

Values express n (%) or mean ± standard deviation.

BMI z-score, age- and sex-specific body mass index z-score; VD, vaginal delivery; CD, cesarean delivery.

^†^ P-values were from t-tests (comparisons of means) and from Chi-square tests of independence (comparison of proportions), and they were all compared with the characteristics of included children.

^‡^ P-values for comparing proportions between VD and CD.

^*^ P-values for comparing proportions between VD and elective CD and non-elective CD.

^**^ Sample sizes for “children excluded” and “children having <4 anthropometric measures” were 713 and 337, respectively, because there were seven children with missing birth weight data.

**Table S2.** Multivariable-adjusted odds ratios (OR) and 95% confidence intervals (CI) for offspring BMI z-score trajectories associated with cesarean delivery as well as its subtypes vs. vaginal delivery by sex.

| Variable | Increasing from moderate to high | Increasing from mild to moderate | Increasing from low to high | Stable low |
| --- | --- | --- | --- | --- |
| **Boys** |  |  |  |  |
| VD | 1.00 (Reference) | 1.00 (Reference) | 1.00 (Reference) | 1.00 (Reference) |
| CD (both elective and non-elective combined) | 1.62 (1.01-2.60) | 0.93 (0.70-1.24) | 1.62 (1.11-2.35) | 0.83 (0.56-1.21) |
| Elective CD | 1.65 (1.01-2.70) | 0.93 (0.65-1.34) | 1.70 (1.07-2.69) | 0.75 (0.51-1.09) |
| Non-elective CD | 1.59 (0.98-2.58) | 0.92 (0.65-1.30) | 1.59 (1.03-2.45) | 1.25 (0.51-1.09) |
| **Girls** |  |  |  |  |
| VD | 1.00 (Reference) | 1.00 (Reference) | 1.00 (Reference) | 1.00 (Reference) |
| CD (both elective and non-elective combined) | 1.60 (1.01-2.56) | 0.93 (0.69-1.24) | 1.59 (1.10-2.29) | 0.88 (0.60-1.28) |
| Elective CD | 1.63 (1.00-2.66) | 0.93 (0.65-1.33) | 1.79 (1.15-2.78) | 0.71 (0.49-1.04) |
| Non-elective CD | 1.58 (0.99-2.54) | 0.95 (0.67-1.34) | 1.47 (0.97-2.23) | 1.26 (0.89-1.76) |

Models were adjusted for gestational age at birth, birth weight, maternal age at birth, maternal education level, maternal BMI at the first prenatal visit, parity, hypertensive disorders of pregnancy, and gestational diabetes mellitus.

BMI z-score, age- and sex-specific body mass index z-score; VD, vaginal delivery; CD, cesarean delivery.

All of the four BMI z-score trajectory groups were compared to the “stable mild” group.
